# Poor Vision from Copper Deficiency

**DOI:** 10.1007/s13668-025-00712-6

**Published:** 2025-11-21

**Authors:** Leslie M. Klevay

**Affiliations:** University of North Dakota School of Medicine and Health Sciences, 1301 North Columbia Rd, Grand Forks, ND 58202-9037 USA

**Keywords:** Copper deficiency, Diagnostic tests, Dietary copper, Optic neuropathy, Vision loss

## Abstract

**Purpose:**

A dozen medical articles describe poor vision from copper deficiency. They are collected here along with some ideas about copper nutrition, epidemiology, patient evaluation and treatment.

**Recent findings:**

Clinical descriptions have been brief. Severe vision loss of a middle-aged woman improved from 20/400 to 20/25 with copper replacement for 10 months.

**Summary:**

The optimal chemical form, dose, duration or route of copper repletion are defined poorly. Copper gluconate is the preferred supplement; several mg of elemental copper usually should be given daily for months. Superoxide dismutase in erythrocytes is one of the more sensitive tests of deficiency.

## Introduction

A dozen medical articles describe poor vision from copper deficiency. Only a minority was published in journals related to ophthalmology, rather they appeared in journals on gastroenterology, hematology, internal medicine etc. One was published in a journal related to trace elements. They are collected here along with some ideas about copper nutrition, epidemiology, patient evaluation and treatment.

## Clinical Findings

Clinical descriptions have been brief [1]. Gregg et al. mentioned optic neuritis [2]. Spinazzi et al. noticed optic nerve involvement [3]. Naismith et al. described a woman with an acute onset of blindness [4]. Khaleeli et al. described a man with reduced visual acuity [5]. Pineles et al. found progressive optic neuropathy [6]. Copper (and iron and zinc) was decreased in blood and hair of children with night blindness [7]. Yarandi et al. mentioned major vision loss associated with optic neuropathy [8]. Harrison et al. described progressive vision loss over two years in a boy eating a limited, monotonous diet [9] (my interpretive comments about copper can be found at the electronic site of that journal).

Shah and Tamhankar restored progressive vision loss of a woman by replenishing copper [10]. Rapoport and Lavin improved severe vision loss of a woman from 20/400 to 20/25 with copper replacement for 10 months [11]. Neither measurement error nor spontaneous remission seems likely.

### Human Copper Deficiency

A new and severe neuropathy is being found increasingly. It resembles that of pernicious anemia, but it responds to copper rather than vitamin B_12_. It may be as common as vitamin B_12_ deficiency and may be very important in its differential diagnosis [12]. Ten to 15 cases have been reported from single clinics [13, 14]. Poor balance is the most frequent complaint. No one knows what percent of deficient people have poor vision.

If copper deficiency is suspected and obvious causes such as bariatric or other gastrointestinal surgery, dental adhesives high in zinc, hemochromatosis, iron or zinc supplementation, lead poisoning, malabsorption and soft drink excess have been excluded, dietary deficiency may be the cause [15]. At least one fourth of adults consume less copper than the estimated average requirement published for the United States and Canada [16].

Effects of copper deficiency on eyes seems to have been overlooked, generally, but careful reading of Kumar reveals mention of myelo-opticoneuropathy [17]. Jaiser and Winston suggest that visual impairment in clioquinol intoxication may be from impaired copper metabolism [18].

Copper deficiency has been studied for nearly a century [19]. Examination of classical texts reveals little information about eyes or vision (e.g., Owen [20]). The phenomena described here seem to be newly recognized. Decreased myelination of optic nerves has been found in deficient animals [21, 22]. Myelination is a copper dependent process [23, 24].

## Patient Evaluation

Rapoport and Lavin provide some of the more complete, medical data on this phenomenon [11]. I suggest that patients be evaluated by both a neurologist and an ophthalmologist adept in nutriture assessment. The former will detect ataxia, poor balance, demyelination, and tremor; the latter will find decreased color vision, night blindness, optic neuropathy, and pale optic nerves, etc. Initially positive findings should be reevaluated on completion of copper therapy.

Several laboratory tests have been used to assess copper status [25]. Superoxide dismutase in erythrocytes seems most sensitive to copper depletion. Others can be ranked in decreasing order: enzymatic ceruloplasmin, serum copper, immunoreactive ceruloplasmin. Potentially more sensitive tests include extracellular superoxide dismutase, leucocyte copper, platelet cytochrome c oxidase, and serum lysyl oxidase [16].

Nine medical articles show that serum copper increases if C-reactive protein is elevated; correlation coefficients range from 0.29 to 0.79 [26]. Plasma (or serum) copper may be an insensitive test of deficiency; numerous experiments with animals reveal that plasma copper may be normal or increased even though copper in liver or other organs is low [1]. Liver copper probably is the best index of copper nutriture [27].

## Treatment with Copper

The optimal chemical form, dose, duration or route of copper repletion are defined poorly [11, 17]. If possible, anti-copper agents such as extra zinc should be discontinued. Longer trials with higher doses are preferred. If suspicion is high and diagnostic tests are equivocal, therapeutic trial may be warranted considering the large improvement of the patient above. Toxicity of copper is low, although intravenous copper must be given slowly.

Signs and symptoms related to the nervous system from copper deficiency sometimes respond slowly and incompletely to copper supplementation [3, 8, 17]. Abnormal hematology, if present, usually resolves quickly. It is not clear whether low cure rates result from insufficient supplementation or severe deficiency. Nerves grow slowly and re-myelination also may be slow [1].

A few thousand middle-aged patients were studied for more than six years to determine possible benefit of dietary supplements on vision. Odds of developing Age Related Macular Degeneration were reduced by supplements containing zinc [28]; no one received zinc without also receiving copper. Perhaps copper supplementation rather than zinc was beneficial [1].

Copper gluconate is the only copper supplement listed by the United States Pharmacopeial Convention; cupric oxide, contained in some vitamin-mineral supplements, should be avoided as it is utilized poorly [15]. Supplemental doses of elemental copper effective in several copper depletion/repletion experiments ranged from 2 to 6 mg daily [16]. As the subjects of these experiments had normal physiology, 4–6 mg probably are needed if intestinal physiology has been altered by surgery, etc. Long term therapy may be necessary. The Tolerable Upper Intake Level for copper is 10 mg daily; it is not meant to apply to individuals who are receiving copper under medical supervision [29]. It is unlikely that occasional consumption of foods high in copper such as legumes, mushrooms, dark chocolate, nuts or liver (in order of increasing copper) will prevent this poor vision because the patients probably have an unusually high dietary requirement for copper.

## Conclusion

A new neuropathy from copper deficiency is being found increasingly; it resembles that of pernicious anemia. A dozen scattered, medical articles describing poor vision from copper deficiency are collected. Descriptions of clinical findings have been brief. Potential patients should be evaluated by both a neurologist and an ophthalmologist. Because plasma copper is rather insensitive, newer tests such as superoxide dismutase in erythrocytes should be used. Oral copper, as gluconate, should be taken for long periods.

## Key References

- L.M. Klevay, Ocular lesions from copper deficiency, Indian J Med Res 2017;146(3):430–431. 
  - Notes the brevity of clinical descriptions.
- Y. Rapoport, P.J. Lavin, Nutritional optic neuropathy caused by copper deficiency after bariatric surgery, J Neuro-ophthalmol 2016;36(2):178 − 81.
  - Spectacular improvement in vision from copper supplementation is described.
- N. Kumar, Copper deficiency myelopathy (human swayback), Mayo Clin Proc 2006;81(10):1371-84. 
  - Includes vision in a new, deficiency neuropathy.
- L.M. Klevay, Improving accuracy of normal serum copper, J Trace Elem Med Biol 2016;34:38.
  - Describes the effect of high C-reactive protein on serum copper.
